# Meta-analysis of the prognostic and clinical value of tumor-associated macrophages in adult classical Hodgkin lymphoma

**DOI:** 10.1186/s12916-016-0711-6

**Published:** 2016-10-17

**Authors:** Baoping Guo, Hong Cen, Xiaohong Tan, Qing Ke

**Affiliations:** Department of Chemotherapy, Affiliated Tumor Hospital of Guangxi Medical University, Nanning, People’s Republic of China

**Keywords:** Tumor-associated macrophages, Classical Hodgkin lymphoma, Meta-analysis

## Abstract

**Background:**

The prognostic significance of tumor-associated macrophages (TAM) in adult classical Hodgkin lymphoma (cHL) remains controversial. Here, we report a meta-analysis of the association of CD68 and CD163 infiltration on the clinical outcome of adult cHL.

**Methods:**

A comprehensive search to identify relevant articles was performed in PubMed, Embase, and Google Scholar on January 31, 2016. Using the fixed effect or random effects model of DerSimonian and Laird, hazard ratios (HR) or odds ratios (OR) with 95 % confidence intervals (CIs) were used as the effect size estimate.

**Results:**

Twenty-two eligible studies with a total of 2959 patients were identified. Our analysis indicated that a high density of CD68^+^ TAMs in the tumor microenvironment of adult cHL predicted poor overall survival (OS) (HR: 2.41; 95 % CI, 1.92–3.03), shorter progression-free survival (PFS) (HR: 1.78; 95 % CI, 1.45–2.18), and poor disease-specific survival (HR: 2.71; 95 % CI, 1.38–5.29). High density of CD163^+^ TAMs in the tumor microenvironment of adult cHL also predicted poor OS (HR: 2.75; 95 % CI, 1.58–4.78) and poor PFS (HR: 1.66; 95 % CI, 1.22–2.27). In addition, we demonstrated that a high density of either CD68^+^ or CD163^+^ TAMs was associated with the presence of Epstein-Barr virus in neoplastic cells (OR_CD68_: 3.13; 95 % CI, 2.02–4.84; OR_CD163_: 2.88; 95 % CI, 1.55–5.34). A high density of either CD68^+^ or CD163^+^ TAMs tend to be associated with a more advanced clinical stage (OR_CD68_: 1.25; 95 % CI, 0.93–1.67; OR _CD163_: 1.19; 95 % CI, 0.86–1.63), B-symptoms (OR_CD68_: 1.35; 95 % CI, 0.90–2.01; OR_CD163_: 2.19; 95 % CI, 0.96–5.03), higher International Prognostic Factors Project Score (OR_CD68_: 1.20; 95 % CI, 0.67–2.15; OR_CD163_: 2.00; 95 % CI, 0.92–4.35), and bulky disease (OR_CD68_: 1.47; 95 % CI, 0.88–2.47; OR_CD163_: 1.19; 95 % CI, 0.72–1.96).

**Conclusions:**

Our analyses suggest that a high density of either CD68^+^ or CD163^+^ TAMs is a robust predictor of adverse outcomes in adult cHL. Increased TAMs should be taken into account to further improve prognostic stratification and the planning of appropriate therapeutic strategies.

## Background

Classical Hodgkin lymphoma (cHL) carries an excellent prognosis for most patients, with more than 80 % of patients experiencing long-term remission following conventional chemotherapy or radiotherapy-based protocols [1–3]. However, approximately 20 % of patients experience relapse or disease that is refractory to all conventional therapies, and many patients may suffer short- and long-term treatment-related complications [4–6]. The International Prognostic Factors Project Score (IPS) is the most widely used prognostic system; however, it is rarely employed to modify treatment [7, 8]. Robust prognostic indicators are thus needed to better risk-stratify patients at diagnosis.

cHL is unique among the lymphomas because malignant cells are heavily outnumbered by reactive cells in the tumor microenvironment, such as macrophages, T cells, B cells, eosinophils, mast cells, and other stromal elements. In an adult cHL microenvironment, malignant Hodgkin-Reed-Sternberg cells express a variety of cytokines and chemokines. This is thought to be the driving force behind an abnormal immune response, perpetuated by additional factors secreted by recruited reactive cells in the microenvironment [9]. Steidl et al. [10] were the first to show, by gene expression profiling followed by immunohistochemistry assay, that the quantity of tumor-associated macrophages (TAMs) predicts progression-free survival (PFS). However, most studies that used various antibodies to stain TAMs were inconclusive regarding the association of CD68^+^ and CD163^+^ TAM density and survival. Some studies have indicated that a high density of either CD68^+^ or CD163^+^ TAMs in the tumor microenvironment of adult cHL is associated with poorer outcomes [10–22]. Other studies have not confirmed this finding [23–31], and using TAMs as a biomarker to risk-stratify patients remains controversial.

The objective of this study was to evaluate the prognostic significance of elevated density of CD68^+^ and CD163^+^ TAMs in the tumor microenvironment on overall survival (OS) and PFS in patients with adult cHL. In addition, the relationship between CD68^+^ and CD163^+^ TAMs in the tumor microenvironment of adult cHL and other clinical characteristics was also examined.

## Methods

The present meta-analysis was performed in accordance with the Preferred Reporting Items for Systematic Reviews and Meta-Analyses Statement [32].

### Literature search

We performed a systematic electronic search in PubMed, Embase, and Google scholar for articles published before January 31, 2016. We identified studies by using Medical Subject Heading (MeSH) terms and corresponding keywords, including “macrophages”, “tumor-associated macrophage”, “tumor-infiltrating macrophage”, “intratumoral macrophage”, “TAMs”, “Hodgkin disease”, “classical Hodgkin lymphoma”. No language restriction was applied. We also manually checked the bibliographies of previous reviews and references in all selected studies. Investigators were contacted and asked to supply additional data when key information relevant to the meta-analysis was missing.

### Selection criteria

Studies were included if they (1) were prospective or retrospective cohort studies or clinical trials; (2) contained immunochemistry data used to evaluate TAM by anti-CD68 or anti-CD163; (3) were studies that involved patients with a proven diagnosis of cHL performed to investigate the correlation of CD68^+^ and CD163^+^ TAM density and survival; or (4) tumor-associated macrophages in adult cHL were described as high (above the cut-off value) and low (below the cut-off value) density. Studies were excluded if they were (1) review articles, case reports, animal or in vitro studies; or (2) analyzing pediatric patients or serum CD68 or CD163 samples.

### Study selection, data extraction, and end points

Two investigators (BPG and HC) independently selected articles and extracted data from eligible studies. Disagreements were resolved by consensus. Baseline characteristics and outcomes were extracted from the selected articles. Information taken from each study included the name of the first author, year of publication, country, number of patients, stage, sex, treatment, WHO subtype, Epstein-Barr virus (EBV) status, antibodies, thresholds, median follow-up, and outcome correlation. In situ hybridization (ISH) analysis for EBV-encoded RNA (EBER) was performed in all included studies. Among the included studies, there were six studies using a follow-up time of less than 5 years [13, 16, 23, 26, 27, 29]. We chose OS and PFS as endpoints for our meta-analysis. Various endpoints for PFS were reported in the selected studies, including event-free survival (EFS [12, 14, 18, 19, 22]) and failure-free survival (FFS) [15]. As PFS, EFS, and FFS had a similar definition in these articles, we operationally defined PFS to include EFS or FFS for studies that did not provide PFS.

### Quality assessment

The Newcastle-Ottawa Scale (NOS) was used to assess the quality of each individual study; this was performed independently by two authors (BPG and XHT). The NOS comprises three quality parameters: selection (0–4 points), comparability (0–2 points), and outcome assessment (0–3 points). Studies with NOS scores of ≥ 6 were determined to be high-quality [33].

### Statistical analysis

The primary outcome was survival in patients with a high density of either CD68^+^ or CD163^+^ TAMs compared to those with a low density of CD68^+^ or CD163^+^ TAMs. The cut-off value for “high versus low” CD68^+^ or CD163^+^ TAM density was determined by the investigators in each study. Hazard ratios (HRs) with 95 % confidence intervals (CIs) were combined to obtain an effective value. For studies in which HRs and CIs were not available, we used the method proposed by Parmar et al. [34] to derive estimates from survival curves. An HR > 1 indicated poor survival in the group with high CD68^+^ or CD163^+^ TAM density.

For the pooled analysis of the relationship between high CD68^+^ or CD163^+^ TAM density and EBV status or other clinical parameters (such as stage), odds ratios (OR) and their 95 % CIs were combined to give the effective value. An OR > 1 indicated a higher probability that EBV was present and advanced stage in the group with high CD68^+^ or CD163^+^ TAM density. The point estimate of the HR or OR was considered statistically significant at the *P* < 0.05 level if the 95 % CI did not include the value 1. Heterogeneity was assessed by the χ^2^ test and expressed as *I* ^*2*^ index [35], which describes the percentage of total variation across studies due to heterogeneity rather than chance (25 % low heterogeneity, 50 % medium, 75 % high). If heterogeneity existed between primary studies, a random effects model was used. Otherwise, a fixed effects model was used in meta-analysis [36]. If results of both univariate and multivariate Cox regression analyses were reported, multivariate models were used for a more accurate estimate of the effect of CD68 or CD163 expression. Begg’s test [37] and Egger’s test [38] were used to detect possible publication bias. All analyses were carried out using STATA statistical software package version 12.0 (STATA, College Station, TX).

## Results

### Selection and characteristics of studies

**Fig. 1:**
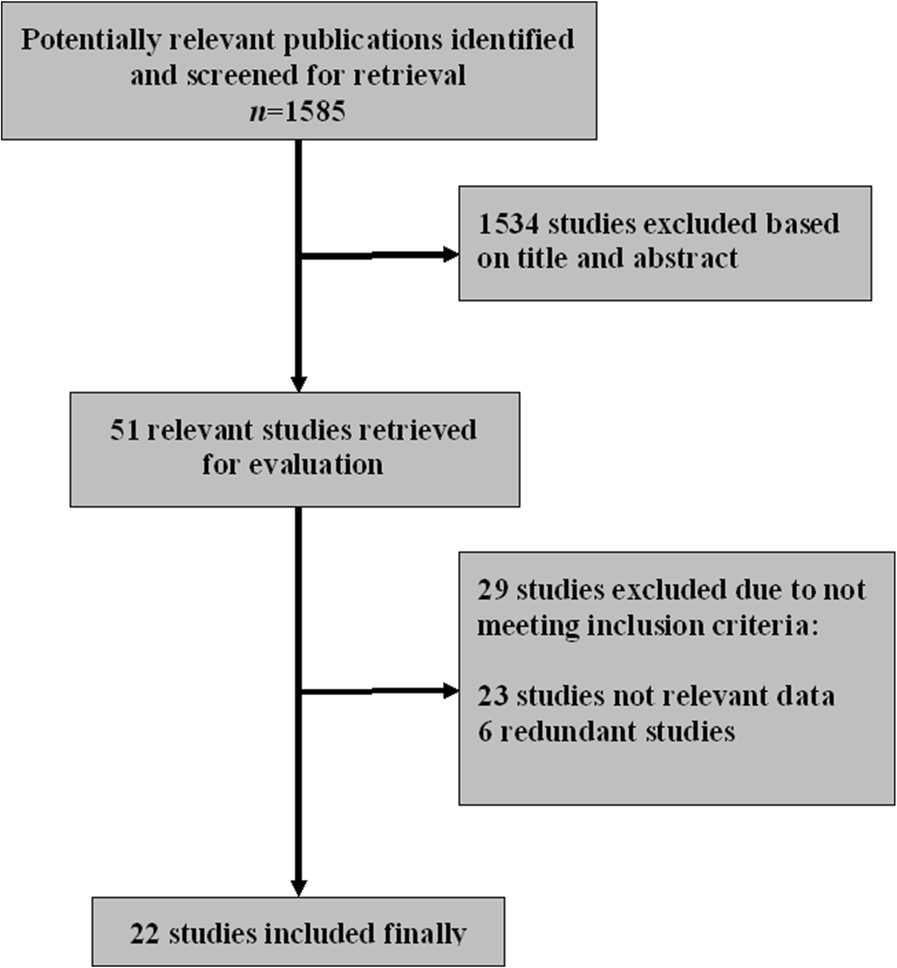
Flow diagram of the systematic review and meta-analysis process

Our initial search yielded 1585 articles. After removing duplicates and screening the titles and abstracts, 31 articles were reviewed in further detail. After reviewing the full text, 22 unique studies were selected as potentially appropriate for inclusion in the meta-analysis [10–31]. Our search strategy is presented in Fig. 1.

The main characteristics of the included studies are summarized in Table 1. Sixteen studies were retrospective cohorts and six were prospective cohorts. Studies were published between 2010 and 2016. The studies were conducted in 12 countries (Denmark, Italy, Switzerland, France, Spain, Israel, United Kingdom, Canada, United States, Japan, South Korea, Egypt, India, Serbia, and China). Population sizes ranged from 61 to 288, with a total of 2959 patients. The reported median or mean age ranged from 21 to 54 years across eligible studies.

**Table 1:** Characteristics of studies included in the meta-analysis

Study, Year	Country	Number of subjects	Stage	Treatment, n (%)	WHO subtypes, *n* (%)	Hodgkin-Reed-Sternberg EBV status, *n* (%)	Antibodies (clone)	Scoring	Threshold(s)	Follow-up median (range) & mean (range) (in years)	Outcome correlation
Steidl et al., 2010 [10]	Canada	166	Limited/advanced	ABVD ± RT 165 (99), RT alone 1 (1)	NS 140 (84), MC 11 (7), Others 5 (3), NOS 10 (6)	Neg 137 (84), Pos 27 (16)	CD68 (KP1)	Visual estimation	5 %, 25 %	4.0 (0.5–20.8)	CD68, adverse (PFS, DSS)
Tzankov et al., 2010 [11]	Switzerland	105	Limited/advanced	ABVD ± RT 30 (28), COPP ± RT 48 (46), RT alone 27 (26)	NS 60 (57), MC 32 (30), Others 5 (5), NOS 8 (8)	Neg 85 (81), Pos 20 (19)	CD68 (PGM1)	Visual, cell counting	0.82 %	11.8 (1.0–27.6)	CD68, adverse (OS)
Kamper et al., 2011 [12]	Denmark	288	Limited/advanced	ABVD/COPP ± RT, ABVD ± RT, RT alone	NS 237 (82), MC 47 (16), NOS 4 (1)	Neg 193 (67), Pos 95 (33)	CD68 (KP1), CD163 (10D6)	Computer-assisted point counting	7.8 % (CD68), 21.1 % (CD163)	7.0 (0.2–18.6)	CD68, adverse (EFS, OS); CD163, adverse (EFS, OS)
Hohaus et al., 2011 [13]	Italy	93	Limited/advanced	ABVD ± RT 55 (59), BEACOPP ± RT 32 (34), MOPP/Other ± RT 6 (7)	NS 64 (69), MC 4 (4), Other 6 (8), NOS 19 (20)	Neg 37 (69), Pos 17 (31)	CD68 (PGM1)	Visual estimation	5 %	1.1 (0.1–6.8)	CD68, adverse (PFS)
Zaki et al., 2011 [23]	Japan	82	Limited/advanced	ABVD ± RT, RT alone	NS 20 (24), MC 52 (63), Other 10 (12)	NR	CD68 (PGM1), CD163 (10D6)	Visual, cell counting in 0.146 mm^2^	Median (CD68), Median (CD163)	Mean: 4.0 (0.8–9.6)	CD68, NSS (OS); CD163, adverse (OS)
Azambuja et al., 2012 [24]	USA	265	Limited/advanced	ABVD ± RT 265 (100)	NS 180 (68), MC 52 (20), Others 18 (6), NOS 15 (6)	Neg 122 (58), Pos 87 (42)	CD68 (KP1), CD163 (10D6)	Visual estimation	5 %, 25 % (CD68), 5 %, 25 % (CD163)	6 (1.5–11.7)	CD68, NSS (PFS, DSS); CD163, NSS (PFS, DSS)
Yoon et al., 2012 [14]	South Korea	144	Limited/advanced	ABVD 113 (79), C-MOPP 10 (7), ABVD/C-MOPP hybrid 15 (10), BEACOPP 6 (4)	NS 90 (63), MC 34 (24), Others 11 (7), NOS 9(6)	Neg 66 (46), Pos 78 (54)	CD68 (KP1), CD163 (10D6)	Visual estimation	20 % (CD68), 20 % (CD163)	5.4 (0.7–19.0)	CD68, adverse (EFS, OS); CD163, adverse (EFS, OS)
Tan et al., 2012 [15]	Canada	287	Advanced	ABVD ± RT 144 (50), Stanford V ± RT 143 (50)	NS 223 (78), MC 38 (13), Others 9 (3), NOS 17(6)	Neg 238 (83), Pos 49 (17)	CD68 (KP1), CD163 (10D6)	Computer-assisted image analysis	12.7 % (CD68), 16.8 % (CD163)	5.5	CD63, adverse (FFS, OS); CD163, adverse (FFS, OS)
Sanchez-Espiridion et al., 2012 [31]	USA	103	Advanced	NR	NS 74 (73), MC 22 (22), Others 6 (6)	NR	CD68 (KP1 and PGM1), CD163 (10D6)	Computer-assisted point counting	5 %, 25 %, median (CD68), 5 %, 25 %, median (CD163)	NR	CD68, NSS (FFS, OS); CD163, NSS (FFS, OS)
Abdou et al., 2013 [16]	Egypt	61	Limited/advanced	Chemotherapy	NS 33 (54), MC 20 (33), Others 8 (13)	NR	CD68 (KP1)	Visual, cell counting	40 % (CD68)	Mean: 1.2 ± 1.7	CD68, adverse (OS)
Greaves et al., 2013 [17]	United Kingdom	122	Limited/advanced	Anthracycline-based ± RT 56 (46), Alkalator-based ± RT 52 (43), RT alone 14 (11)	NS 93 (78), MC 25 (20), Others 2 (2)	Neg 84 (69), Pos 38 (31)	CD68 (KP1)	Computer-assisted image analysis	5 %, 15 %	16.5 (2–40)	CD68, adverse (FFTF, OS) across the 3 defined groups
Deau et al., 2013 [25]	France	59	Limited/advanced	ABVD 47 (80), EBVP 8 (14), BEACOPP 3 (5)	NS 54 (92), Non-NS 5 (8)	NR	CD68 (KP1)	Visual estimation	25 %	NR	CD68, adverse (PFS); CD68, NSS (OS)
Panico et al., 2015 [26]	Italy	121	Limited/advanced	ABVD ± RT 101 (83), ABVD-like ± RT 20 (17)	NS 73 (60), MC 40 (33), Others 8 (7)	NR	CD68 (KP1)	Visual, cell counting	30 per 0.023 mm^2^	Mean: 3.5 (0.1–9.3)	CD68, adverse (OS); CD68, NSS(PFS)
Casulo et al., 2013 [18]	USA	81	Limited/advanced	ABVD 38 (47), Stanford V 11(13), ABVD/MOPP 16 (20), others 16 (20)	NR	NR	CD68 (KP1)	Computer-assisted image analysis	30 %	8.8	CD68, adverse (OS)
Koh et al., 2014 [19]	South Korea	116	Limited/advanced	ABVD ± RT 116 (100)	NS 78 (67), MC 22 (19), Others 8 (7), NOS 8 (7)	Neg 73 (63), Pos 43 (37)	CD68 (KP1), CD163 (10D6)	Visual estimation	20 % (CD68), 20 % (CD163)	6.2 (3.8–10.3)	CD68, adverse (EFS, DSS, OS); CD163, adverse (EFS, DSS, OS)
Ping et al., 2014 [27]	China	72	Limited/advanced	ABVD 49(68), BEACOPP 13(18), others 10 (14)	NS 41(57), MC 23 (32), Others 8 (11)	NR	CD68 (KP1)	Visual estimation	250/HPF	3.9 (0.7–15)	CD68, NSS (OS);
Klein et al., 2014 [28]	USA	88	Limited/advanced	ABVD ± RT 88 (100)	NS 55 (63), MC 6 (7), Others 1 (1), NOS 19 (22)	NR	CD68 (KP1), CD163 (10D6)	Visual estimation	5 %, 25 % (CD68), 5 %, 25 % (CD163)	NR	CD68, NSS (OS); CD163, adverse (OS) 25 % threshold
Touati et al., 2014 [20]	France	158	Limited/advanced	ABVD 102(65), ABVD/MOPP 26 (17), BEACOPP 11 (7), ABVD-like 9 (5), others 10 (6)	NS 130 (82), MC 21 (13), Others 7 (5)	Neg 106 (67), Pos 40 (25), Not done 12 (8)	CD68 (PGM1)	Visual estimation	25 %	5.5 (0.2–16.2)	CD68, adverse (PFS, OS)
Kayal et al., 2014 [30]	India	100	Limited/advanced	ABVD ± RT 88 (88), EVAP RT 11 (11), Other 1 (1)	NS 51 (51), MC 47 (47), Others 2 (2)	NR	CD68 (CD68/G2)	Visual, cell counting	12.9 %, 18.2 %, 25 % (the quartiles)	5.7	CD68, NSS (PFS, DSS)
Agur et al., 2015 [29]	Israel	98	Limited/advanced	ABVD 60(60), BEACOPP 29(29), others 9 (1)	NS 33 (34), MC 7 (7), Others 58 (59)	NR	CD68 (PGM1)	Visual, cell counting	25 %	Mean: 3.8 (0.9–7.8)	CD68, NSS (PFS)
Moreno et al., 2015 [21]	Spain	249	Advanced	NR	NS 162 (65), MC 68 (27), Others 19 (8)	NR	CD68 (PGM1), CD163 (10D6)	Computer-assisted point counting	30 %	NR	CD68, adverse (OS)
Jakovic et al., 2016 [22]	Serbia	101	Advanced	ABVD ± RT 101 (100)	NS 80 (79), MC 13 (13), Others 8 (8)	NR	CD68 (PGM1)	Visual estimation	25 %	8.6 (0.2–16)	CD68, adverse (EFS, OS)

*ABVD* doxorubicin, bleomycin, vinblastine, and dacarbazine, *BEACOPP* bleomycin, etoposide, doxorubicin, cyclophosphamide, vincristine, procarbazine, and prednisone, *COPP* cyclophosphamide, vincristine, procarbazine, and prednisone, *C-MOPP* cyclophosphamide, vincristine, procarbazine, prednisone, *EVAP* etoposide, vinblastine, adriamycin and prednisolone, *EBVP* epirubicin, bleomycin, vinblastine and prednisone, *MOPP* mustargen, oncovin, procarbazine, and prednisone, *RT* radiotherapy, *Stanford V* vinblastine, doxorubicin, vincristine, bleomycin, mustard, etoposide, and prednisone, *NS* nodular sclerosis, *MC* mixed cellularity, *Neg* negative, *Pos* positive, *RT* radiotherapy, *NOS* not otherwise specified, *PFS* progression-free survival, *DSS* disease-specific survival, *EFS* event-free survival, *OS* overall survival, *FFS* failure-free survival, *FFTF* freedom from treatment failure, *NR* not reported, *NSS* not statistically significant, *HPF* high power field

The points of study quality assessed on the NOS for assessing quality ranged from 3 to 9 (mean = 6.23), with higher values indicating better methodology. Certainly, the low quality studies were also included in the analyses. The results of this quality assessment are shown in Table 2.

**Table 2:** Assessment of the risk of bias in each cohort study using the Newcastle–Ottawa scale

Study		Selection (0–4)			Comparability (0–2)		Outcome (0–3)			Total
	REC	SNEC	AE	DO	SC	AF	AO	FU	AFU	
Steidl et al. [10]	1	1	1	1	1	1	1	1	1	9
Tzankov et al. [11]	1	1	1	1	0	0	0	1	1	6
Kamper et al. [12]	1	1	1	1	1	1	1	1	0	8
Hohaus et al. [13]	1	1	1	1	0	0	1	0	0	5
Zaki et al. [23]	0	1	1	1	0	0	0	1	0	4
Azambuja et al. [24]	1	1	1	1	1	1	1	1	0	8
Yoon et al. [14]	1	1	1	1	1	0	1	1	1	8
Tan et al. [15]	1	1	1	1	1	1	1	1	1	9
Sanchez-Espiridion et al. [31]	0	1	1	1	0	0	0	1	0	4
Abdou et al. [16]	0	1	1	1	0	0	0	0	0	3
Greaves et al. [17]	1	1	1	1	1	1	1	1	1	9
Deau et al. [25]	0	1	1	1	0	0	0	1	0	4
Panico et al. [26]	1	1	1	1	0	0	1	1	0	6
Casulo et al. [18]	1	1	1	1	1	0	1	1	0	7
Koh et al. [19]	1	1	1	1	0	0	1	1	0	6
Ping et al. [27]	0	1	1	1	0	0	1	1	0	5
Klein et al. [28]	0	1	1	1	0	0	1	1	1	6
Touati et al. [20]	1	1	1	1	0	0	0	1	1	6
Kayal et al. [30]	1	1	1	1	0	0	1	1	0	6
Agur et al. [29]	1	1	1	1	0	0	1	1	1	7
Moreno et al. [21]	0	1	1	1	0	0	1	1	0	5
Jakovic et al. [22]	1	1	1	1	0	1	0	1	0	6

“1” indicates that the study has satisfied the item and “0” indications the opposite

*REC* representativeness of the exposed cohort, *SNEC* selection of the non-exposed cohort, *AE* ascertainment of exposure, *DO* demonstration that outcome of interest was not present at start of study, *SC* study controls for age, sex, *AF* study controls for any additional factors (Chemotherapy, radiotherapy), *AO* assessment of outcome, *FU* follow-up long enough (36 M) for outcomes to occur, *AFU* adequacy of follow-up of cohorts (≥90 %)

### Prognostic significance of CD68^+^ TAMs

**Fig. 2:**
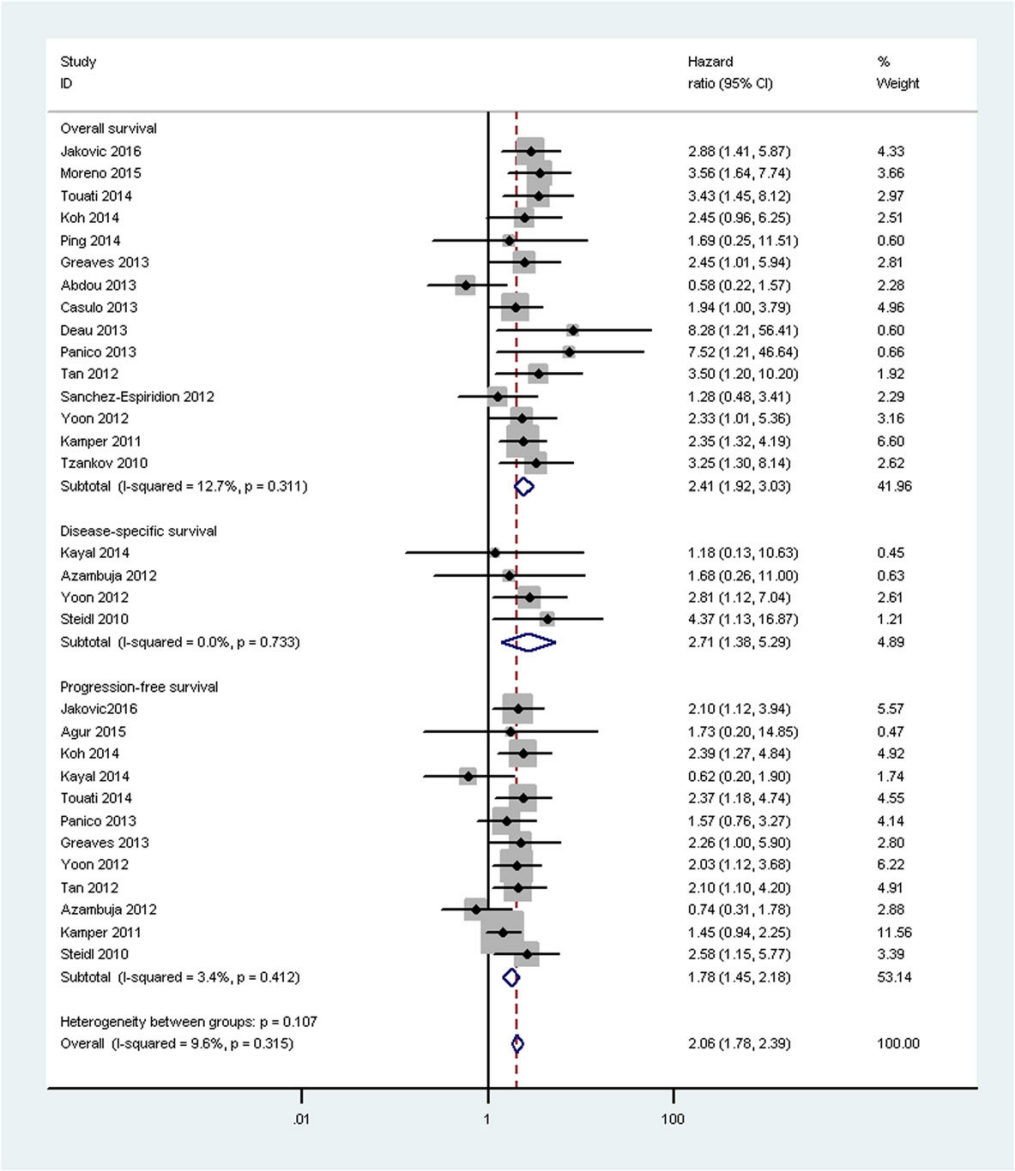
Meta-analysis of the hazard ratios for progression-free survival, disease-specific survival, and overall survival for a high versus a low CD68^+^ tumor-associated macrophage (TAM) density. Hazard ratios (HRs) and 95 % confidence intervals (CIs) from individual studies are depicted as squares and horizontal lines, respectively. The pooled estimate is shown as a diamond shape, where the center represents the pooled HRs and the horizontal borders represent the 95 % CI. HRs are defined as high CD68^+^ versus low CD68^+^ TAM density; therefore, a hazard ratio > 1 represents a higher risk of death or progression associated with a high CD68^+^ TAM density

Fifteen studies were included in the analysis of CD68^+^ TAMs on OS in adult cHL. The results of our meta-analysis showed that a high CD68^+^ TAM density was associated with shorter OS than a low CD68^+^ TAM density, with a pooled HR of 2.41 (95 % CI, 1.92–3.03). No significant heterogeneity was found across the studies (*I* ^*2*^ = 12.7 %, *P* = 0.31). Twelve studies reported data on CD68^+^ TAMs and PFS in adult cHL. Meta-analyses results demonstrated that a high CD68^+^ TAM density was associated with shorter PFS than a low CD68^+^ TAM density, with a pooled HR of 1.78 (95 % CI, 1.45–2.18). No significant heterogeneity was found across the studies (*I* ^*2*^ = 3.4 %, *P* = 0.41). HRs for disease-specific survival (DSS) were available in four studies. The estimated pooled HR showed that a high CD68^+^ TAM density was associated with poorer DSS than a low CD68^+^ TAM density, with a pooled HR of 2.71 (95 % CI, 1.38–5.29). No significant heterogeneity was found across the studies (*I* ^*2*^ = 0.0 %, *P* = 0.73; Fig. 2).

### Prognostic significance of CD163^+^ TAMs

**Fig. 3:**
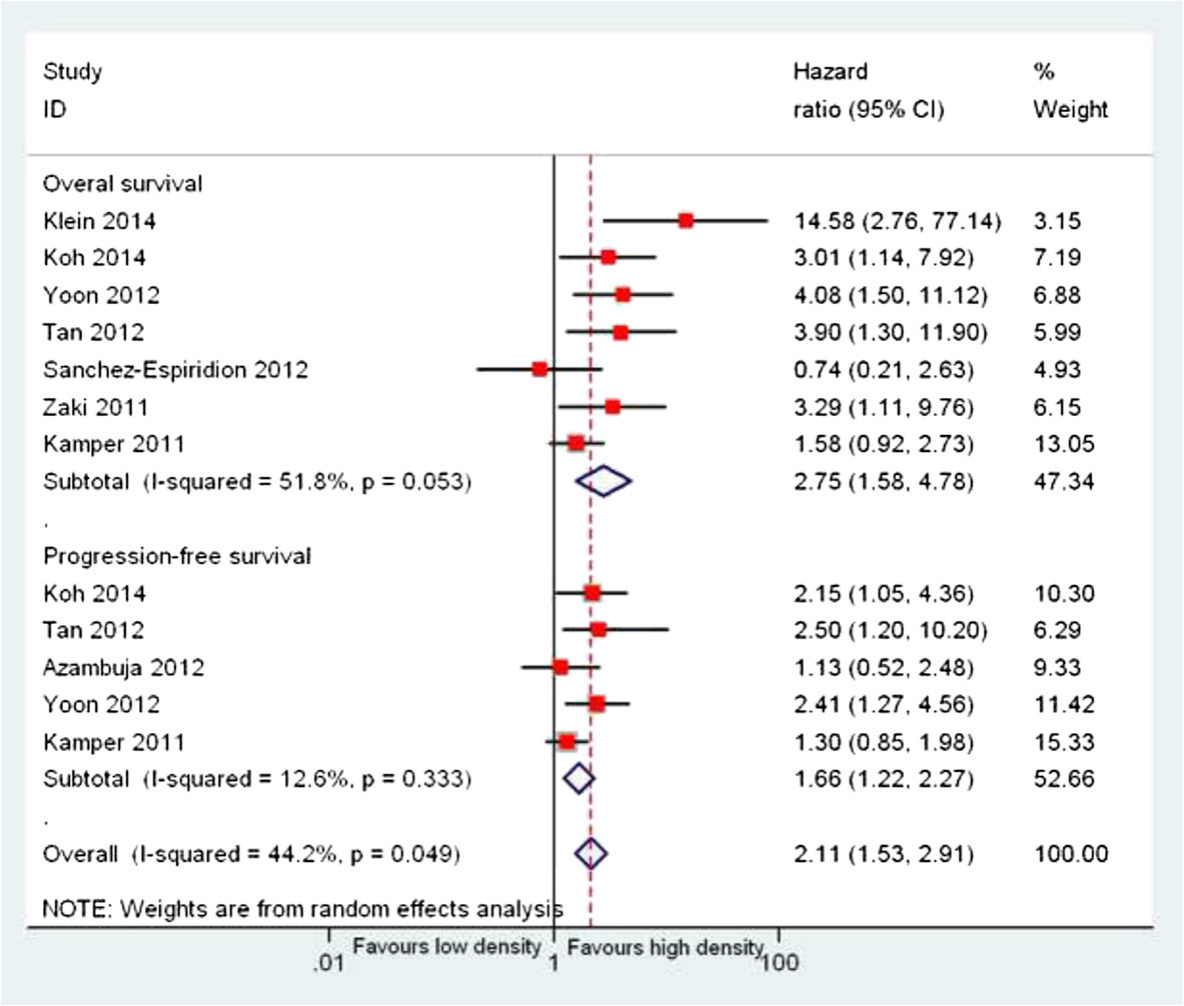
Meta-analysis of the hazard ratios for progression-free survival and overall survival for high CD163^+^ versus low CD163^+^ tumor-associated macrophage (TAM) density. Hazard ratios and 95 % confidence intervals for death or progression associated with high versus low CD163^+^ TAM density

Meta-analysis of seven studies showed poorer OS in the high CD163^+^ TAM density group than in the low CD163^+^ TAM density group, with a pooled HR of 2.75 (95 % CI, 1.58–4.78). There was an indication of medium heterogeneity across the studies, but it did not reach statistical significance (*I* ^*2*^ = 51.8 %, *P* = 0.053). HRs for PFS were available in five studies with adult classical Hodgkin lymphoma. The results of our meta-analysis showed that high CD163^+^ TAM density was associated with shorter PFS than low CD163^+^ TAM density, with a pooled HR of 1.66 (95 % CI, 1.22–2.27). No significant heterogeneity was found across the studies (*I* ^*2*^ = 12.6 %, *P* = 0.11; Fig. 3). Notably, only one study provided relevant data on the correlation of CD163^+^ TAMs with DSS; therefore, the pooled analysis could not be performed.

### CD68^+^ and CD163^+^ TAMs and clinical features

**Fig. 4:**
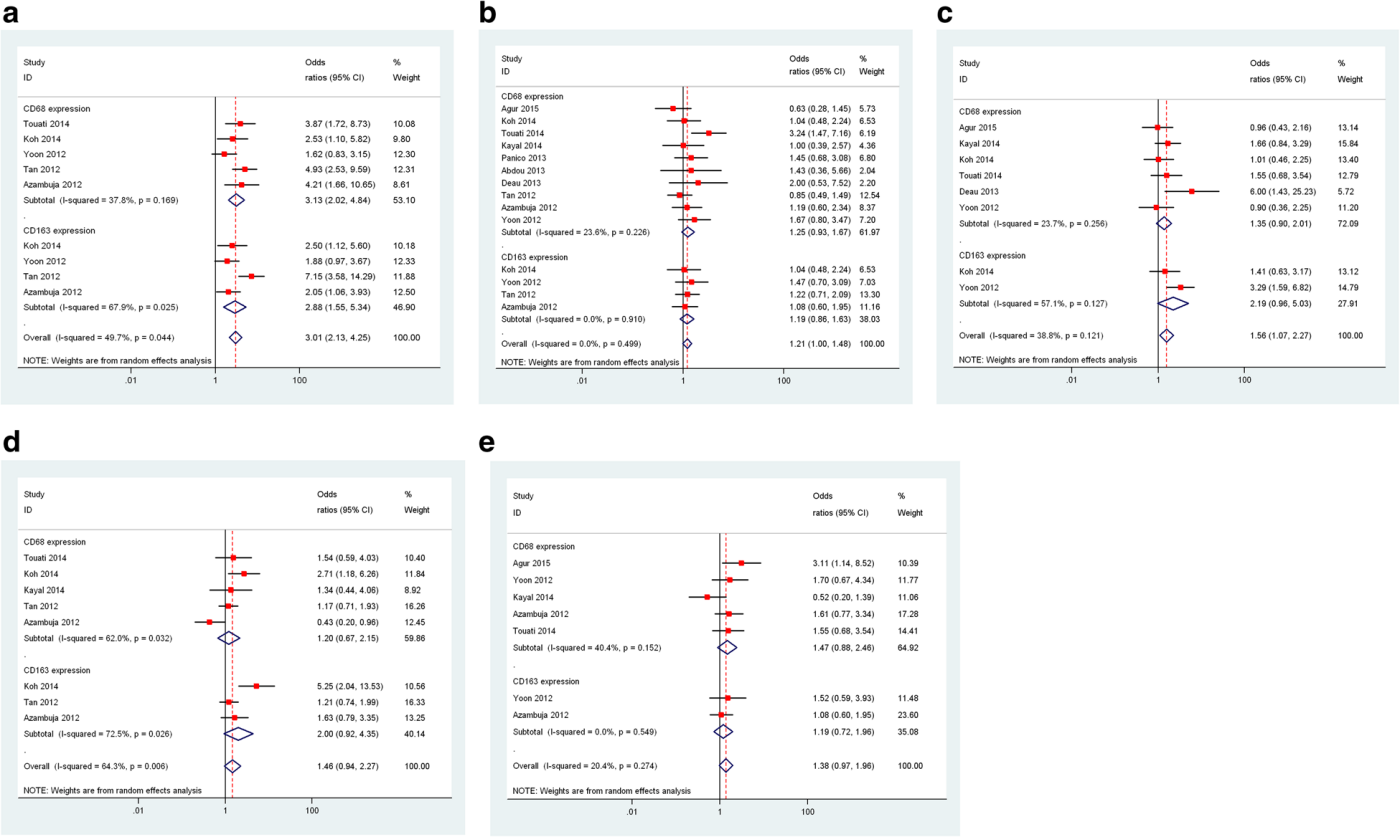
Meta-analysis of the association between CD68^+^ and CD163^+^ tumor-associated macrophage (TAM) density and clinical features. The Forest plots show (**a**) the association between a high CD68^+^ and CD163^+^ TAM density and presence of Epstein-Barr virus of adult classical Hodgkin lymphoma (cHL); (**b**) the association between a high CD68^+^ and CD163^+^ TAM density and presence of Ann Arbor stage of adult cHL; (**c**) the association between a high CD68^+^ and CD163^+^ TAM density and B-symptoms of adult cHL; (**d**) the association between a high CD68^+^ and CD163^+^ TAM density and International Prognostic Factors Project Score of adult cHL; and (**e**) the association between a high CD68^+^ and CD163^+^ TAM density and bulky disease of adult cHL

Meta-analysis of five studies showed a high CD68^+^ TAM density was associated with the presence of EBV, with a pooled OR of 3.13 (95 % CI, 2.02–4.84). There was an indication of slight heterogeneity across the studies, but it did not reach statistical significance (*I* ^*2*^ = 37.8 %, *P* = 0.17). The results of meta-analysis of the four studies showed a correlation between high CD163^+^ TAM density and the presence of EBV. Because significant heterogeneity was found across the studies (*I* ^*2*^ = 67.9 %, *P* = 0.03), a pooled OR of 2.88 (95 % CI, 1.55–5.34) was calculated on the basis of a random-effects model (Fig. 4a).

Ten studies reported data on CD68^+^ TAMs and Ann Arbor stage in adult cHL. Meta-analysis of these studies found a trend for a correlation between high CD68^+^ TAM density and advanced stage, with a pooled OR of 1.25 (95 % CI, 0.93–1.67). No significant heterogeneity was found across the studies (*I* ^*2*^ = 23.6 %, *P* = 0.23). Four studies reported data on CD163^+^ TAMs and Ann Arbor stage in adult cHL. Meta-analysis of the four studies showed a trend for a correlation between high CD163^+^ TAM density and advanced stage, with a pooled OR of 1.19 (95 % CI, 0.86–1.63). No significant heterogeneity was found across the studies (*I* ^*2*^ = 0.0 %, *P* = 0.91; Fig. 4b).

Six studies reported data on CD68^+^ TAMs and B-symptoms in adult cHL. The result of meta-analysis of the six studies showed a trend for a correlation between a high CD68^+^ TAM density and B-symptoms, with a pooled OR of 1.35 (95 % CI, 0.90–2.01). No significant heterogeneity was found across the studies (*I* ^*2*^ = 23.7 %, *P* = 0.26). Two studies reported data on CD163^+^ TAMs and B-symptoms in cHL. The result of meta-analysis of the two studies showed a trend for a correlation between high CD163^+^ TAM density and B-symptoms, with a pooled OR of 2.19 (95 % CI, 0.96–5.03). No significant heterogeneity was found across the studies (*I* ^*2*^ = 57.1 %, *P* = 0.12; Fig. 4c).

Five studies reported data on CD68^+^ TAMs and IPS in adult cHL. The result of meta-analysis of the five studies showed a trend correlation between a high CD68^+^ TAM density and higher IPS, with a pooled OR of 1.20 (95 % CI, 0.67–2.15). Three studies reported data on CD163^+^ TAM density and IPS in adult cHL. The result of meta-analysis of the three studies showed a trend for a correlation between high CD163^+^ TAM density and a higher IPS, with a pooled OR of 2.00 (95 % CI, 0.92–4.35). Significant heterogeneity was found across the studies (*I* ^*2*^ = 62.0 % for CD68^+^ TAMs and *I* ^*2*^ = 72.5 % for CD163^+^ TAMs, all *P* < 0.05; Fig. 4d).

Five studies reported data on CD68^+^ TAMs and bulky disease in adult cHL; meta-analysis revealed a trend correlation between a high CD68^+^ TAM density and bulky disease, with a pooled OR of 1.47 (95 % CI, 0.88–2.47). Two studies reported data on CD163^+^ TAMs and bulky disease in adult cHL. The result of meta-analysis of the two studies showed a trend for a correlation between a high CD163^+^ TAM density and bulky disease, with a pooled OR of 1.19 (95 % CI, 0.72–1.96). No significant heterogeneity was found across the studies (*I* ^*2*^ = 40.4 % for CD68^+^ TAM density and *I* ^*2*^ = 0.0 % for CD163 expression, all *P* > 0.10; Fig. 4e).

### Publication bias

**Fig. 5:**
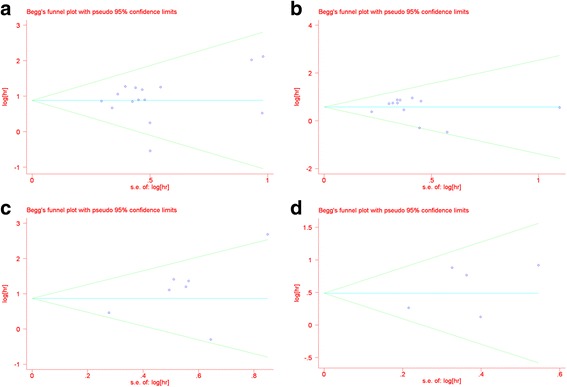
Begg’s funnel plot showed no publication bias among the included studies. **a** CD68^+^ tumor-associated macrophages (TAMs) and overall survival OS (*P* = 0.488). **b** CD68^+^ TAMs and PFS (*P* = 0.732). **c** CD163^+^ TAMs and OS (*P* = 0.230). **d** CD163^+^ TAMs and PFS (*P* = 1.000)

A more formal evaluation of CD68^+^ TAMs using Begg’s and Egger’s tests showed no evidence of significant publication bias (OS, Begg’s test *P* = 0.488; Egger’s test *P* = 0.522; shown in Fig. 5a; PFS, Begg’s test *P* = 0.732; Egger’s test *P* = 0.639; shown in Fig. 5b). For CD163^+^ TAMs, there was no evidence for significant publication bias (OS, Begg’s test *P* = 0.230; Egger’s test *P* = 0.172 (Fig. 5c); PFS, Begg’s test *P* = 1.000; Egger’s test *P* = 0.356 (Fig. 5d)).

## Discussion

Many recent studies have focused on the impact of non-neoplastic cells on disease pathobiology, specifically immunohistochemical typing of cells in the microenvironment, with the goal of identifying potential prognostic markers and therapeutic targets in adult cHL. Due to flawed statistical analyses and an inability to validate findings, few biomarkers have translated into clinical practice. Meta-analysis is a valuable tool in biomarker validation; thus, in this study, we carried out a meta-analysis to examine the correlation between TAMs and adult cHL prognosis.

This is the first meta-analysis investigating survival of cHL patients in which the survival of patients with high and low density of CD68^+^ and CD163^+^ TAMs was compared. Our results demonstrated that a high density of either CD68^+^ or CD163^+^ TAMs in the tumor microenvironment significantly predicted poor OS and shorter PFS for adult cHL (*P* = 0.000 and *P* = 0.000, respectively). A high CD68^+^ TAM density was associated with worse DSS than that in the group with a low CD68^+^ TAM density (*P* = 0.004). Furthermore, we also conducted a pooled analysis on the correlation between macrophage-associated markers and adult cHL EBV positivity. This finding suggests that a high density of either CD68^+^ or CD163^+^ TAMs is strongly correlated with EBV positivity (*P* = 0.000 and *P* = 0.001, respectively). In addition, these results suggested a trend towards a high density of both CD68^+^ and CD163^+^ TAMs with the presence of B-symptoms, advanced stage, bulky disease, and an IPS greater than 3; however, these results were not statistically significant. Taken together, the results of our pooled analysis support that a higher density of either CD68^+^ or CD163^+^ TAMs in the tumor microenvironment of adult cHL is associated with a higher risk of worst outcome.

The results of our meta-analysis indicate an association between increased density of both CD68^+^ and CD163^+^ TAMs in the tumor microenvironment of adult cHL and poor outcome. Among the included studies, different score methods and threshold values were used in the measurement of CD68^+^ and CD163^+^ TAMs using immunohistochemistry. Most of the studies used manual visual scoring techniques, ranging from cell counting [11, 23, 24] to computer-assisted methods of point counting [12, 21] and image analysis [15, 17]. The lack of consistent and reproducible of thresholds in these studies made it difficult to separate patients into low- and high-risk populations. Reproducibility of data produced in different laboratories and of assay methods for tissue sections will need to be a focus of future studies. Few biomarkers have translated into clinical practice since the reproducibility of immunohistochemical scoring has been suggested as a reason for inconclusive results, and thus more robust multigene predictors have been reported based on expression profiling [10]. However, these gene expression studies were limited by small case numbers and the lack of available clinical data.

In addition, the antibody used to stain macrophages varied. It has been reported that the KP1 clone does not only react specifically with macrophages, but also reacts with myeloid and fibroblastic cells [39]. The clone 10D6 for CD163 is more specific for macrophages than either the KP1 or PGM1 clones for CD68 [40]. Thus, CD163 may be a better marker for TAMs than CD68. When antibodies to CD68 and CD163 have both been used, some studies have noted discrepancies in the association of macrophages with patient outcomes. Martin-Moreno et al. [21] observed an association between increased CD68-stained cells and DSS in a cohort, but no association was observed using an antibody against CD163. In contrast, Zaki et al. [23] and Klein et al. [28] only saw an association between CD163 and outcome. Some studies indicate that TAMs in adult cHL promote tumor growth and angiogenesis, suppression of adaptive immune responses, and contribute to immune evasion by tumor cells, and thus may be associated with poor prognosis [41–44].

These analyses have some advantages and important implications. First, study quality scores, assessed using the NOS, had a mean score of 6.23, giving validity to the results of the present meta-analysis. Second, Egger’s test did not detect publication bias, indicating that the results are not biased. Third, this study shows that a high density of both CD68^+^ and CD163^+^ TAMs is associated with poorer outcome, which suggests that the TAMs may be useful as a drug therapeutic target. Fourth, our study identifies a subgroup of adult cHL tumors with poorer outcome. Finally, it highlights the importance of the development of an accurate biomarker for assessment of adult cHL.

The meta-analysis performed in this study had several limitations. First, negative studies are less frequently published, or are published with less detailed results, making them less assessable, potentially leading to some bias. Second, our meta-analysis is based on data from trials from which the results have been published, and we did not obtain updated individual patient data. Use of individual patient data may further enhance the accuracy and reduce the uncertainty of the estimates. Third, because of the variety of endpoints reported in adult cHL studies, we operationally defined adult cHL PFS to include EFS or FFS in studies that did not provide PFS. Fourth, some of the HRs with 95 % CIs were extracted from the Kaplan–Meier survival curve. Finally, among the included studies in the current meta-analysis, six had a follow-up time of less than 5 years, which may have incorporated bias.

## Conclusions

In conclusion, the results presented here indicate that a high density of either CD68^+^ or CD163^+^ TAMs in the tumor microenvironment of adult cHL is associated with poor survival. A high density of both CD68^+^ and CD163^+^ TAMs was associated with the presence of EBV in neoplastic cells, and might provide essential information for the prediction of advanced stage, B-symptoms, bulky disease, and higher IPS. Evaluation of TAMs should be considered in prospective clinical trials, and patients with increased TAMs may benefit from more intensive chemotherapy or novel agents designed to disrupt crosstalk between Hodgkin-Reed-Sternberg cells and benign macrophages.
